# Histological Comparison of Two Cryopeeling Methods for Photodamaged Skin

**DOI:** 10.1155/2014/950754

**Published:** 2014-03-12

**Authors:** Janyana Deonizio, Betina Werner, Fabiane A. Mulinari-Brenner

**Affiliations:** ^1^Dermatology Department, Federal University of Parana, Rua General Carneiro, 181 Alto da Glória, 80060-900 Curitiba, PR, Brazil; ^2^Pathology Department, Federal University of Parana, Rua General Carneiro, 181 Alto da Glória, 80060-900 Curitiba, PR, Brazil

## Abstract

*Background*. Cryopeeling is a technique that uses cryotherapy not only on actinic keratoses lesions, but also all over the photodamaged skin. *Objectives*. To investigate the histological changes induced by two cryopeeling methods (liquid nitrogen (LN) and portable system (PS)). *Methods*. Sixteen patients (*n* = 16) with multiple actinic keratoses on the forearms were treated with cryopeeling technique using LN for one forearm and PS for the other, randomly. Skin biopsies were taken before and after the procedures. *Results*. There was no statistical difference between the epidermal and Grenz zone thicknesses or density of elastic fibers after treatments. The amount of melanin pigment was lower after PS treatment (*P* < 0.05). In a blind analysis of paired pre- and postprocedure slides, it was not possible to identify cases which underwent treatment, both in global analysis of quality of the skin and in specific analysis (considering only the aspect of stratum corneum). *Discussion*. The results indicate the inconsistency of histological improvement after treatments, and, likely, since the method causes superficial exfoliation, a reliable marker was not found in the analysis. *Conclusions*. Despite cosmetic benefits on photodamaged skin and efficient treatment of actinic keratoses lesions, cryopeeling was not able to induce measurable histological changes in solar elastosis, epidermal organization, or epidermal and Grenz zone thicknesses. One should keep in mind the possibility of hypopigmentation risk of the method.

## 1. Introduction

Cryotherapy using liquid nitrogen (LN) is the most common form of treatment of actinic keratosis [1]. It is an easy access option for dermatologists with good cost benefit in the treatment of these lesions [2–4]. A portable system (PS) is a more recent alternative for cryotherapy, which uses gases (dimethylether, propane, or isobutane) in a portable plastic container. In this method the tip may achieve a temperature of −55°C. Using LN, whose boiling point is −196°C, the temperature to be achieved in the tissue for the treatment of malignancies is between −50°C and −60°C [5]. Cryopeeling is a technique that uses cryotherapy in a diffuse manner throughout the skin region affected by sun damage in order to promote cell renewal and desquamation, with possible benefits in the appearance of new lesions caused by photodamage. Few studies were performed evaluating cryopeeling technique until now [3, 6, 7]. The results obtained with cryopeeling treating actinic keratoses were satisfactory and more effective than 5-fluorouracil. It seems that cryopeeling is efficient, affordable, and easy to apply [3]. Despite the fact that focal ablative methods are considered effective options for actinic keratoses, the treatment of the entire “field of cancerization” has guided the treatment of multiple actinic keratoses. The field of cancerization is defined by the entire photodamaged area that, although being without clinical lesions, demonstrates precancerous changes and genetic mutations, which precede tumor development. In this context, cryopeeling is a technique that uses cryotherapy diffusely throughout the skin of the area affected by sun damage with possible benefits in the development of new lesions. The aim of this study was to investigate and compare the possible histological changes induced by two cryopeeling methods: LN and PS.

## 2. Methodology

**Figure 1:**
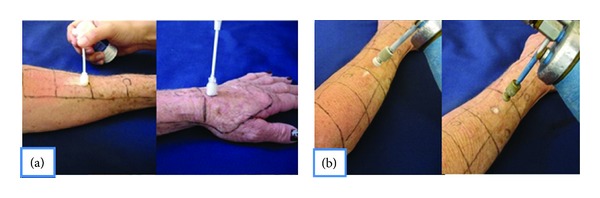
Patients received the treatment of cryopeeling using the PS for one forearm (a) and using LN for the other forearm (b), randomly. The cryopeeling was performed by applying the freezing substance with brush movements along the extension of the forearm until the area was frozen. After this, actinic keratoses lesions were individually treated.

Sixteen patients were selected (*n* = 16) during dermatology appointments who presented with considerable photodamage and multiple actinic keratoses on upper limbs. We included men and women between 50 and 80 years of age (mean age 68.5 ± 9.6 years). Patients with decompensated chronic diseases or coagulation disorders were excluded. The study was approved by the Ethics Committee of the Hospital of the Federal University of Parana given the current official standards (Resolution CNS 196/96, Law 6.638/79, and Normative Resolution 04/97). Patients received the treatment of cryopeeling using the PS for one forearm and using LN for the other forearm, randomly. We applied an occlusive topical anesthetic (lidocaine 5% and prilocaine 5%) two hours before the procedure. To guide application, quadrants and demarcation of actinic keratoses lesions to be individually treated were made. The cryopeeling was performed by applying the freezing substance with brush movements along the extension of the forearm until the area was frozen [7]. After this, actinic keratoses lesions were individually treated with variable time of freezing set by the investigator (Figures 1(a) and 1(b)). Curettage was previously performed on very hyperkeratotic lesions. According to the manufacturer's guidelines for the use of PS, the valve was pressed until a few drops of the product came out from the tip. Then the applicator was rotated 90° and stayed about fifteen seconds until the tip was frozen. The tip has been slid with rotational movements along the skin causing the transitory bleaching (Figure 1(a)). In the day following the procedure patients used Vaseline topically to moisturize the skin and reduce discomfort of healing.

## 3. Histological Analysis

A 4 mm skin biopsy punch was performed in all patients on each forearm, at the lateral region, before and 60 days after cryopeeling. Actinic keratoses lesions were intentionally avoided and the second sample was taken in the same region but 1 cm apart from the first biopsy in order to avoid the scaring area. Random codes were established for all cases in order to allow for blind analysis of data in all histological evaluations regarding the time of biopsy (before or after treatment) and which method was used (LN or PS). At the end of the study each patient had performed four skin biopsies. The specimens were fixed in 10% formalin and submitted to routine histological processing. Microscopic sections were made for hematoxylin and eosin (H&E), Fontana Masson, and Weigert stains. Several parameters were analyzed as described below, by the same observer at the same day.

### 3.1. Hematoxylin and Eosin

**Figure 2:**
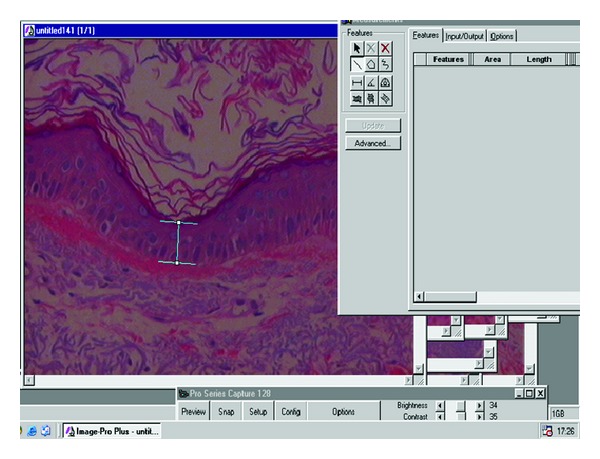
Evaluation of epidermal thickness using the average of consecutive measurements of the three thinnest areas found.

We analyzed the thickness of the epidermis and Grenz zone (area of loose papillary dermis spared by elastosis) using the Image-Pro software program version 4.5.123. The thickness of the epidermis was calculated by using the three smallest measurements found (Figure 2).

The Grenz zone thickness measurement was obtained by the average of the three thickest measurements. The largest was discarded when it was more than twice compared to the second largest measure and replaced by a fourth measure.

Blindly, a dermatopathologist analyzed the pairs of slides before and after procedure from each patient. Taking into account a comprehensive global review of the quality of the skin (appearance of the stratum corneum, epidermal thickness, presence of solar lentigines, and quality/quantity of elastosis) it was aimed to establish which slide corresponded to the pretreatment and posttreatment, or if this was not possible to define. A second analysis was performed in the same way, but considering only the analysis of the stratum corneum.

### 3.2. Fontana Masson Staining

We analyzed the distribution of melanin pigment in the basal layer using a weighted average as follows: the total length of the epidermis was divided into 5 segments, each of which corresponded to 20% of the total. Each segment was classified according to the amount of pigment: weak, moderate, or intense corresponding to weights 1, 2, and 3, respectively. Thus, each specimen had a score for pigment distribution. For example: 20% with weak pigment, 20% with moderate pigment, and 60% with intense pigment; (0.2 × 1 + 0.2 × 2 + 0.6 × 3)/6 = score of 0.4.

### 3.3. Weigert Staining

We analyzed the density of solar elastosis in paired slides before and after procedures for each patient, blindly. The aim was to define which slide had a higher density of elastic fibers or if there was no difference between them.

## 4. Statistical Analysis

The student's *t*-test for paired data was used for pre- and posttreatment analysis. For comparison between treatments (LN and PS), the *t*-test for independent samples was used. To analyze the density of elastic fibers and blind paired slides analysis, the Fisher exact test was used.

## 5. Results

We analyzed a total of 16 patients and 31 skin biopsies from 16 forearms treated with LN (*n* = 16) and 15 forearms treated with PS (*n* = 15). One case from PS group was excluded because of technical paraffin inclusion issues. Histological analyses are summarized in Table 1.

Considering the analysis of the epidermal thickness, in 54% of the cases, an increased thickness was demonstrated after treatment. Regarding the Grenz zone thickness, in 52% of the cases, there was an increased thickness after treatment, although for both epidermal and Grenz thicknesses no significant difference was demonstrated after treatments (*P* > 0.05).

Quantification of melanic pigment, considering the weighted average intensity of pigment correlated with the extent of the area analyzed, showed that in 61% of the cases the pigment decreased, in 32% increased, and in 7% remained unchanged. There was a tendency of decrease of pigment intensity with both treatments but this difference was significant only in the group of the PS (*P* = 0.025).

The density of elastic fibers was higher after the procedure in 52% of the cases analyzed after both treatments, but no statistically significant difference was found (*P* > 0.05). There were three cases in which a considerable dermal fibrosis was noted: one before the procedure with LN, one after treatment with LN, and one after treatment with PS.

**Figure 3:**
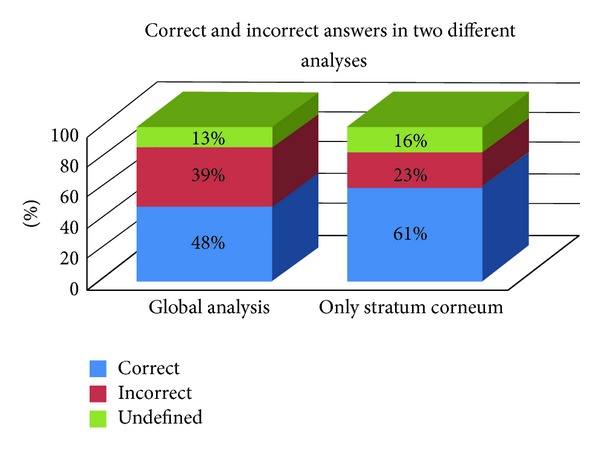
Blind assessment of pre- and postprocedure specimens in order to presume which one underwent treatment. Results are demonstrated by percentage of correct answers, considering a global analysis (stratum corneum, epidermal thickness, presence of lentigines, and elastosis) and a second analysis considering only the aspect of stratum corneum.

In the analysis of H&E slides, both global analysis and specific analysis of stratum corneum did not show significant differences. Trying to presume which specimen was submitted to treatment, the percentage of correct answers was 48% in global analysis and 61.3% in specific analysis of stratum corneum with no statistically significant difference for correct answers (Figure 3).

## 6. Discussion

Although cryotherapy is a method widely used in clinical practice, few studies using cryopeeling technique have been conducted so far [3, 6, 7]. Despite the fact that the focal ablative methods are considered effective options, a more recent concept related to “field of cancerization” treatment has guided the treatment of multiple actinic keratoses. The “field of cancerization” is defined by the entire photodamaged area that, however, does not demonstrate clinical lesions, harbors precancerous changes, and genetic mutations, which precede tumor development. Considering this fact, other treatments can be chosen for the treatment of actinic keratoses as photodynamic therapy and topical medications, in order to treat a larger area and those subclinical changes. 5-Fluorouracil, diclofenac, and topical imiquimod are alternatives following this approach strategy to these lesions [4]. After treatment with photodynamic therapy histological evaluation demonstrated an ability to reduce the histological signs of photodamage, reducing the proliferation marker Ki-67 and p53 marker of early carcinogenesis [8]. Interestingly, despite the improvement of the basal keratinocytes dysplasia, as assessed by scale of two different observers, in 45% of cases, there was still some degree of dysplasia. Following this concept of field of cancerization, cryopeeling technique used in the treatment of actinic keratoses and photodamage would be interesting in order to improve the appearance of skin and, combined with intensive photoprotection, in preventing the appearance of malign lesions [4]. PS is a more recent alternative for cryotherapy, which uses gases (dimethylether, propane, or isobutane) in a portable plastic container. In this method the tip may achieve a temperature of −55°C. With LN, whose boiling point is −196°C, the temperature to be achieved in the tissue for the treatment of malignancies is between −50°C and −60°C [5]. PS may be convenient, since there is no need to be replenished periodically, like LN, and has a long expiration time of three years.

Histological changes due to photoaging are highly debated in the literature. It is believed that there is a decreased cell turnover resulting in reduced turnover rate of the stratum corneum, epidermal atrophy, increased healing time, and less effective scaling. There is an accumulation of corneocytes making the skin surface rougher. Within the dermis, there is a reduction of about 20% of its thickness, with disorganization of collagen fibers and accumulation of abnormal elastin-containing material [9]. The three primary components of the dermis are collagen, elastin, and glycosaminoglycans. With photoaging collagen fibers, which are responsible for skin strength and support, become disorganized and arranged in rope-like bundles. In contrast to the proportion of 80% of type I collagen and 15% of type III collagen in young skin, in aged skin the amount of type III collagen increases [9–11]. Exposure to ultraviolet radiation induces transcription factors involved in gene activation of matrix metalloproteinases and consequent production of collagenases, gelatinases, and stromelysin, perhaps the responsible mechanism for decreased collagen levels [9]. The photodamage is also characterized by elastosis: changes in elastic fibers characterized by the accumulation of amorphous elastin material and thick elastic fibers in the papillary dermis. The initial response to sun damage is hyperplastic, resulting in increased amounts of elastic fibers. However, it is believed that, subsequently, degenerative response takes place resulting in a decrease of the skin elasticity. Additionally, there is a change in the normal pattern of immature oxytalanic elastic fibers located in the papillary dermis. This delicate ascendant network, which extends perpendicularly from the upper section of the dermal papilla until just beneath the basal membrane, gradually tends to disappear. All glycosaminoglycans (hyaluronic acid, dermatan sulfate, and chondroitin sulfate) are decreased in photodamaged skin, specially hyaluronic acid [9].

In photoaging, there is no consensus regarding the epidermis behavior: some studies show thinning of the epidermis and others thickening [9]. Although we have not shown a significant increase in the thickness of the epidermis after treatments, there was a higher tendency for that with LN treatment (*P* = 0.09 compared to *P* = 0.13 in PS). The fact that the LN was more efficient in treating actinic keratoses and more painful compared to the PS, in a previous study [6], suggests that it is a more aggressive method and, therefore, may lead to a greater epidermal response.

Although there was a decrease of the pigment after both treatments, this difference was only significant with the PS. Cryotherapy is a method that is well known to induce hypopigmentation. During the use of the PS for actinic keratoses treatment an unexpected increase of the frozen area was often observed. This fact deserves special attention since it may lead to a high risk of an uncontrolled frozen area which might have contributed to this finding.

Three cases showed dermal fibrosis: one before the procedure, one after LN treatment, and one after PS treatment. Thus one may not attribute this fibrosis to the procedures and a random finding should be considered.

The medium peel with trichloroacetic acid is able to induce an increase of elastic fibers in the papillary dermis after the procedure, establishing what was called Grenz zone [12]. Grenz zone is also a term used to designate the area spared by subepidermal inflammatory infiltrate in granuloma annulare. The real meaning of Grenz is “frontier” and this term was used in this study, by analogy, to designate the area that separates the epidermis from subepidermal area of severe elastosis in the dermis.

Cryopeeling technique is well tolerated and effective in the treatment of actinic keratoses, with clinical benefit evidence in improving the surface appearance of the skin [6]. Despite the evidence of clinical benefits, this was not reflected proportionally on the histological analysis of skin treated with the technique. It was not possible to demonstrate histological changes in solar elastosis, organization, and epidermal thickness or Grenz zone thickness after cryopeeling treatment. The capacity of many procedures to induce histological changes has been evaluated in previous studies, although we agree that the histological analysis is a very challenging task. Firstly there is no standard and reliable protocol in literature to follow in order to analyze the changes induced by some procedure in photodamaged skin. Secondly there is great variation of the samples, even though considering the same patient. Finally, the fixing and staining techniques may provide many other variables. In our study, for histological analysis, all samples were prepared and stained at the same time to minimize stain and fixation variability. Furthermore we tried to minimize errors in the measurements by analyzing the data by a single observer on the same day. Evaluation of epidermal thickness was performed using the average of three consecutive measurements of the thinnest areas found. The aim of using the smallest measurements was to discard the increased epidermal thickness under acrosyringium openings. On the other hand, Grenz zone thickness measure was obtained calculating the average of the three thickest measures. In order to reduce the measurement errors, the largest measure was discarded when it was more than twice compared to the second largest measure and replaced by a fourth one.

In blind assessment of pre- and postprocedure slides in order to presume which one underwent treatment, considering the global analysis of the quality of the skin (stratum corneum, epidermis, lentigines and solar elastosis), it was not being able to identify the cases submitted to treatment accurately (*P* > 0.05). Narrowing the analysis and considering only the changes of the stratum corneum, we obtained a greater number of right answers, although still not statistically significant. The impossibility to predict which sample underwent the procedures indicates the inconsistency, if present, of histological improvement after treatments. The highest density of elastic fibers, although found in many cases after treatment, also failed to establish a statistically significant difference. The fact that the quantitative parameters (epidermal and Grenz zone thicknesses) also have not shown significant differences after treatments corroborates to these findings. Cryopeeling, by definition, is a superficial peeling which cannot reach deeper levels of the dermis. Despite the evident surface appearance and texture improvements in all patients, this was not reflected by measurable histological changes. Maybe the adequate parameter to evaluate the changes was not found. Perhaps the improved appearance of skin was due to the treatment of actinic keratoses themselves and not due to the treatment of entire photodamaged skin.

Furthermore previous studies have demonstrated a hyperplastic response of epidermis after several peeling sections, although only one session with this treatment was not able to induce the same response [12]. In our study, cryopeeling was also performed in a single session, which may be a plausible explanation for the absence of significant difference in histological findings. Considering the lack of histological, or at least unmeasurable benefit, in medium term follow-up, the results also raise a discussion about the real benefit of using cryopeeling under the inherent risk hypopigmentation of the method. Histological evaluation was performed 60 days after the procedure which implies a medium term evaluation of the effects of the technique. Only long term follow-up studies would be able to evaluate the real long term benefit in new lesions development in those patients treated with cryopeeling.

The conclusion of this study was that cryopeeling technique was not able to induce measurable histological medium term changes and has doubtable benefit in the treatment of photodamage despite of the improvement of the superficial aspect of the skin. Besides it should be taken into consideration the possible risk of hypopigmentation using the method.

## Figures and Tables

**Table 1:** Histological findings are summarized. Epidermal and Grenz zone thicknesses values were obtained by average of three measurements. Pigment intensity score was based on a weighted average considering intensity and distribution of pigment. The intensity of pigment decreased after PS treatment (*P* = 0.025).

	Before treatment	After treatment	*n *	*P *
Epidermal thickness average (*μ*m)				
LN	39.34 (SD 7.83)	43.37 (SD 9.40)	16	0.09
PS	38.26 (SD 6.52)	42.28 (SD 8.24)	15	0.13

Grenz zone thickness average (*μ*m)				
LN	61.41 (SD 29.50)	64.37 (SD 18.07)	16	0.71
PS	71.08 (SD 25.98)	80.58 (SD 37.04)	15	0.36

Pigment intensity score				
LN	75.42 (DP 13.27)	71.88 (DP 17.68)	16	0.54
PS	69.55 (DP 11.04)	58.89 (DP 12.39)	15	**0.025**

LN: liquid nitrogen. PS: portable system. SD: standard deviation.
